# A Comparative Study Between Two Support Surfaces for Pressure Ulcer Prevention and Healing in ICU Patients

**DOI:** 10.7759/cureus.8785

**Published:** 2020-06-23

**Authors:** Aikaterini Marvaki, Georgia Kourlaba, Olga Kadda, Georgios Vasilopoulos, Nikoleta Rovina, Antonia Koutsoukou, Anastasia Kotanidou

**Affiliations:** 1 Internal Medicine, National and Kapodistrian University of Athens Medical School, Athens, GRC; 2 Epidemiology, Center of Clinical Epidemiology and Outcomes Research, Athens, GRC; 3 Nursing, Onassis Cardiac Surgery Center, Athens, GRC; 4 Nursing, University of West Attica, Egaleo, GRC

**Keywords:** supportive surfaces, healing, pressure ulcers, intensive care unit, prevention

## Abstract

Objective

The aim of this research was to compare the effectiveness of two mattresses used in intensive care unit (ICU) high-risk patients in terms of pressure ulcers (PUs) prevention and healing.

Materials and Methods

The studied sample consisted of 70 consecutive patients aged 18 to 65 years hospitalized in two ICUs of a general hospital in Athens, Greece. Virtuoso Mattress System (LINET, Slaný, Czech Republic) was used in 35 patients, and standard memory foam mattress was used in the rest of participants. Patients were firstly assessed on enrollment and then every 72 hours in order to record the appearance or not of PUs, location of PUs, and stage of PUs, with the maximum follow-up not exceeding the 21 days. A number of clinical and biochemical factors, medical treatment, and vital signs were also recorded at each time point.

Results

Of the 70 patients, 40 (57.1%) were men, and the mean ± standard deviation age of the sample was 46.1 ± 14.5 years. The most common area of PUs was the buttocks (34.3%) followed by the shoulders (22.3%), with no statistically significant difference detected between the two groups. Moreover, the proportion of patients having PUs at stage 2 or higher was 23.8% on the third day after admission and 61.1% on the sixth day, with no difference detected between the two groups. Cox proportional hazard model revealed that the Virtuoso mattress was associated with almost 56% lower risk of developing PUs compared with standard foam mattress (HR [95% CI]: 0.44 [0.20-0.93]). The percentage of patients healed using the Virtuoso mattress was significantly lower compared with the standard foam mattress at all time points, with the results reaching statistical significance only on the 12th day after admission (7.7% vs. 66.7%; p < 0.05).

Conclusions

The Virtuoso mattress seems to be more effective compared with standard foam mattresses in the prevention of PUs, whereas the standard foam mattresses are more effective in PU healing process.

## Introduction

Pressure ulcers (PUs) are skin injuries that originate in the epithelial tissue developed in intensive care unit (ICU) patients who are bedridden for long periods. Critically ill patients during their ICU stay are more frequently exposed to PU risk factors and finally develop PU [1].

Many studies have underlined the factors associated with the development of PU, including advanced age, nutritional deficiency, longer hospital length of stay, reduced tissue perfusion, use of vasoactive drugs, sedation, and comorbidities such as diabetes mellitus [2-4]. According to the National Pressure Ulcer Advisory Panel (NPUAP), PU incidence in ICU patients varies between 8.8% and 25.1% and contributes to increased healthcare costs [5].

As PUs are strongly associated with prolonged hospitalization and several adverse events such as infections, bundles of care and programs have been described in previous studies aiming at preventing PU development and avoiding the occurrence of adverse events [6].

The cornerstone of PU prevention is the identification of high-risk groups among ICU patients. Prevention actions such as nurse maneuvering and special mattresses should be added to prevention strategies so as to gain the best result [6].

The risk of developing PUs can be evaluated using specific measurement scales of PU risk factors, such as Braden and Norton scales. Efforts to improve the quality of life of ICU patients must be made through various techniques, improving and reducing the extent of damage, and, if possible, achieve their healing. Along with nursing bundles, the literature refers to the various types of supportive surfaces with various mechanisms such as redistributing patient’s total body weight over the maximum body surface area so as to reduce the pressure exerted at various parts of the body and prevent possible tissue damage [7-10].

In Greece, there are a few studies on the effectiveness of specific supportive surfaces such as continuous airflow system compared with standard memory foams mattresses [11].

Aim

The aim of this study was to compare the effectiveness of the Virtuoso Mattress (LINET, Slaný, Czech Republic) with memory foams mattress in the prevention and healing of PUs in adult patients hospitalized in ICUs and being at high risk of developing PUs.

## Materials and methods

Study design

This is an observational prospective study conducted in two ICUs (ICU-A and ICU-B) of a tertiary general hospital in Athens, Greece.

Participants

All consecutive patients admitted to each ICU during the period between December 2016 and May 2017 were eligible for the study. The exclusion criteria were age less than 18 years and over 65 years, length of ICU stay less than 24 hours, and the presence of PUs at ICU admission. Thirty-five patients from each ICU were enrolled in the study. All enrolled patients or their carers provided written informed consent.

Pressure redistribution support

In ICU-A, the Virtuoso Mattress System (a dual-mode support surface that provides active alternating pressure and active constant low pressure-continuous airflow system) was used, whereas in ICU-B, standard hospital therapeutic memory foams mattresses were used that offer pressure redistribution. Healthcare professionals were trained in the use of these mattresses. All patients were given standard PU preventive care that had to be performed in compliance with validated care protocols compliant with the Good Professional Practice Recommendations (i.e., change position every two hours unless otherwise indicated, daily linen change, once a day skin care, exudate management, etc.).

Data collection

Patients were firstly assessed on enrollment and then every 72 hours in order to record the appearance or not of PUs, location of PUs, and stage of PUs using the clinical criteria and national standards of the European Pressure Ulcer Advisory Panel (EPUAP). Moreover, the “Cubbin and Jackson scale”, which consist of 10 items (i.e., age, weight, general skin condition, mental condition, mobility, hemodynamics, respiration, nutrition status, incontinency, hygiene) was used to assess the PU risk at baseline [12]. All healthcare professionals involved in patients’ skin assessment were properly trained. The maximum follow-up did not exceed the 21 days depending on the length of hospitalization or a withdrawal of the study. Apart from skin assessment through physical examination, a number of clinical and biochemical factors, medical treatment, and vital signs such as arterial blood pressure, heart rate, and oxygen saturation were also recorded at each time point. All these data were obtained from hospital informatics system (e.g., laboratory findings) and patients’ medical record.

Endpoints

The primary endpoint criteria were the appearance of PUs during a period of 21 days after enrollment as well as the time to appearance of PUs. The secondary endpoints were the number of PUs at the first appearance and at each time point, the proportion of patients whose PUs were at stage 2 or higher at each time point of assessment, and the proportion of PUs healed at each time point.

Ethics

This study was performed in compliance with the principles of the Declaration of Helsinki and was approved by the Research Ethics Committee of the hospital.

Statistical analysis

Descriptive statistics were used to describe the characteristics of the studied sample and PUs. Continuous normally distributed variables were presented as mean values ± standard deviation, whereas skewed ones were summarized as median and range (minimum, maximum). Categorical variables were presented as absolute and relative frequencies. The chi-square test was used to evaluate the associations between categorical variables. Student’s t-test or Mann-Whitney test was used to assess the association between a continuous variable and the type of mattress, as appropriate.

A survival analysis was conducted to compare the time to appearance of PUs between the two groups (log rank test). The Cox proportional hazard model was used to assess the relationship between the time without PUs and the type of mattress. Multivariate analyses were not conducted since no factors were found to be statistically significantly associated with the type of mattress or outcomes at a univariate level, indicating that no confounders may exist. A probability value of 5% was considered as statistically significant. Data analysis was performed using SPSS, Version 24 (IBM Corp., Armonk, NY, USA).

## Results

Baseline patients’ characteristics

The demographic and baseline clinical characteristics of the studied sample for each group are presented in Table 1. In the total sample, 57.1% (n = 40) were men, with a mean ± standard deviation age of 46.1 ± 14.5 years. Most patients (88.6%) had normal weight and comorbidities (78.6%). The median (range) length of ICU stay was 15.5 (range: 2-78) days. Both groups of patients were found to be comparable in terms of demographic and clinical characteristics, as well as the level of PU risk at the baseline visit (Table 1). The median ICU length of stay was found to be marginally statistically significantly lower in the group with standard hospital mattress memory foam (12 days) compared with the Virtuoso group (24 days; p = 0.083).

**Table 1:** Baseline demographic and clinical characteristics of the sample. P < 0.05 is statistically significant. SD, standard deviation; SBP, systolic blood pressure; HCT, hematocrit; ICU, intensive care unit

Variables	Virtuoso mattresses	Standard foam mattress	p-Value
	Ν (%)	N (%)	
Gender			0.629
Men	19 (54.3)	21 (60.0)	
Women	16 (45.7)	14 (40.0)	
Weight status			0.452
Normal	32 (91.4)	30 (85.7)	
Obese	3 (8.6)	5 (14.3)	
Cubbin and Jackson Scale			0.151
35-40	15 (42.9)	21 (60.0)	
≤34	20 (57.2)	14 (40.0)	
Comorbidities			0.771
Yes	28 (80.0)	27 (77.1)	
No	7 (20.0)	8 (22.9)	
Sedation			0.403
No	10 (28.6)	7 (20.0)	
Yes	25 (71.4)	28 (80.0)	
Only intravenous fluid administration			0.653
No	4 (11.4)	7 (20.0)	
Yes	31 (88.6)	28 (80.0)	
	Mean (SD)	Mean (SD)	
Age (in years)	45 ± 14	47 ± 15	0.519
SBP (mm Hg)	88 ± 18	85 ± 12	0.317
Pulses	87 ± 19	87 ± 21	0.943
HCT	34 ± 8	34 ± 6	0.877
Glucose	139 ± 74	154 ± 85	0.423
Na	141 ± 5	143 ± 7	0.305
K	4.5 ± 0.7	4.5 ± 0.7	0.932
Albumin	3.2 ± 0.9	3.3 ± 0.6	0.632
pH	7.4 ± 0.08	7.4 ± 0.08	0.596
Breathing rate	19.7 ± 5.6	19.0 ± 4.1	0.596
	Median (range)	Median (range)	
ICU length of stay (in days)	24 (2-78)	12 (2-77)	0.083
Urea	38 (16-281)	33 (11-183)	0.362
Creatinine	0.82 (0.25-6.52)	0.64 (0.21-3.11)	0.142

The effectiveness of mattresses

Table 2 presents the association of mattresses with the presence of PUs, the number of PUs, location of PUs, and stage of PUs. The most common area of PUs was the buttocks (34.3%) followed by the shoulders (22.3%) and the coccyx (18.6%), with no statistically significant difference detected between the two groups. Moreover, the proportion of patients having PUs at stage 2 or higher was 23.8% on the third day after admission and 61.1% on the sixth day, with no difference detected between the two groups, as presented in Table 2.

**Table 2:** The association of mattresses with the presence of PUs, the number of PUs, location of PUs, and stage of PUs. P < 0.05 is statistically significant. PU, pressure ulcer; SD, standard deviation

Variable	Virtuoso, n/N (%)	Standard foam mattress, n/N (%)	p-Value
PU appearance at any time during the 21 days of follow-up	15/35 (42.9%)	18/35 (51.4%)	0.473
PU appearance at each time point			
3rd day	6/32 (18.8%)	16/33 (48.5%)	0.011
6th day	8/25 (32.0%)	9/15 (60.0%)	0.083
9th day	11/21 (52.4%)	4/7 (57.1%)	0.827
12th day	8/13 (61.5%)	2/3 (66.7%)	0.867
15th day	5/9 (53.6%)	2/2 (100.0%)	0.237
18th day	4/8 (50.0%)	1/1 (100.0%)	0.556
21st day	2/4 (50.0%)	1/1 (100.0%)	0.600
Location of PUs			
Shoulder	8 (22.9%)	8 (22.9%)	0.999
Buttocks	12 (34.3%)	12 (34.3)	0.999
Coccyx	5 (14.3%)	8 (22.9%)	0.356
Elbow	0 (0%)	2 (5.7%)	0.151
Heel	2 (5.7%)	5 (14.1%)	0.232
PU stage 2 or higher			
3rd day	1 (16.7%)	4 (25.0%)	0.581
6th day	3 (37.5%)	8 (88.9%)	0.043
9th day	3 (27.3%)	3 (75.0%)	0.143
12th day	4 (50.0%)	1 (50.0%)	0.778
15th day	4 (80.0%)	1 (50.0%)	0.524
	Mean ± SD	MEAN ± SD	
Number of PUs			
3rd day	0.2 ± 0.6	0.6 ± 0.7	0.022
6th day	0.3 ± 0.5	1.3 ± 1.4	0.003
9th day	0.4 ± 0.6	1.9 ± 1.8	0.004
12th day	0.9 ± 0.9	2.5 ± 0.7	0.047
15th day			
Number of PUs at first appearance	1.13 ± 0.35	1.22 ± 0.43	0.517

**Figure 1:**
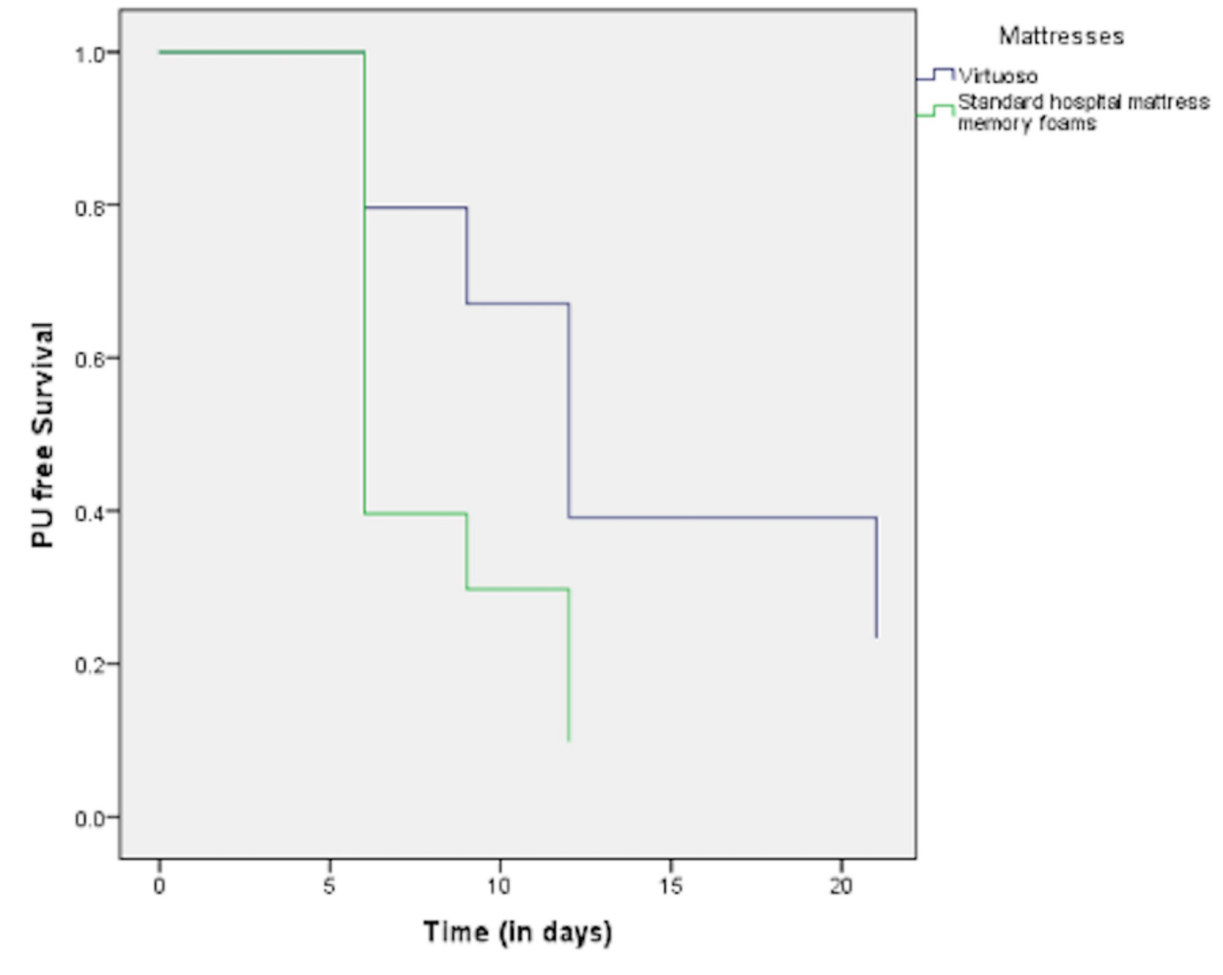
Association between mattress and time to first appearance of PU: Kaplan-Meier curve. PU, pressure ulcer

The comparison of the proportion of patients who developed PU at each time point of follow up between the two groups revealed no difference at all time points with the exception of the time point “three days after ICU admission”, with this proportion being significantly higher in patients with standard hospital mattress memory foams (48.5%) compared with the Virtuoso group (18.8%; p = 0.011). This finding is confirmed by the Kaplan-Meier curves showing that the probability of being free from PUs decreases more rapidly in the standard hospital mattresses memory foams group compared with the Virtuoso one (p = 0.009; log rank test) (Figure 1). Cox proportional hazard model revealed that the Virtuoso mattress was associated with almost 56% lower risk of developing PUs compared with standard hospital mattress memory foams (HR [95% CI]: 0.44 [0.20-0.93]).

Finally, it was detected that the number of PUs was statistically significantly higher in the group with standard hospital mattress memory foam compared with the group with Virtuoso mattress at each time point (Table 2).

With respect to the healing of PUs, the percentage of patients healed using the Virtuoso mattress was significantly lower compared with the standard foam mattress at all time points, with the results reaching statistical significance only on the 12th day after admission (7.7% vs. 66.7%; p < 0.05) (Table 3).

**Table 3:** The effectiveness of mattresses in terms of PU healing. P < 0.05 is statistically significant. PU, pressure ulcer

Variable	Virtuoso, n/N (%)	Standard foam mattress, n/N (%)	p-Value
Healing			
6th day	2/25 (8.0%)	4/15 (26.7%)	0.109
9th day	4/11 (36.4%)	3/6 (50.0%)	0.644
12th day	1/13 (7.7%)	2/3 (66.7%)	0.016
15th day	3/6 (50.0%)	2/2 (100.0%)	0.464
18th day	3/6 (50.0%)	2/1 (100.0%)	0.999
21st day	3/6 (50.0%)	2/2 (100.0%)	0.400

## Discussion

The objective of this study was to assess the effectiveness of two different types of mattresses in the prevention of PU development and PU healing in patients hospitalized in ICUs in Greece. Study findings indicate that in a group of critically ill patients with no Pus, the use of the Virtuoso mattress prevents PU development when compared with standard memory foam mattress. On the other hand, the use of standard memory foam mattresses seems to be more effective in patients who have already developed PUs.

Our findings are in agreement with those of other studies [13-16] conducted in the last decades to compare different types of mattresses in terms of PU prevention and healing. The authors of these studies support that patients managed with air flow mattresses had a significantly lower incidence of PU than those managed with a standard hospital mattress.

For example, Park and Park [17] in their prospective randomized controlled trial compared a viscoelastic foam overlay (VEFO) with a standard hospital mattress for PU prevention. Their studied sample consisted of 110 participants (55 in each group) hospitalized in an ICU of a medical center in South Korea. Skin was assessed daily over a period of two weeks. Their findings suggest that the PU development was significantly lower in patients managed with a VEFO as compared with those managed with a standard hospital mattress (p = 0.001).

A similar randomized clinical trial conducted by Bueno de Camargo et al. [18], aiming to analyze whether a viscoelastic mattress support (intervention group) surface can reduce the incidence of stage II PUs compared with a standard hospital mattress (control group) with pyramidal overlay in critically ill patients, found that the frequency of PU was higher in the control group compared with the intervention group.

In van Leen et al.’s [13] single-center prospective crossover trial on PU incidence in nursing home residents, a static air overlay mattress, without a pump, on top of a viscoelastic foam mattress was compared with a viscoelastic foam mattress alone. A total of 41 patients with no PU were divided into two groups: 21 patients received a viscoelastic foam mattress (control group) and 20 patients received a static air overlay on top of a viscoelastic foam mattress (intervention group) for a period of six months. According to the results of the study, static air overlay mattresses provided a better prevention than viscoelastic foam mattresses alone.

In contrary to the results of our study, Ozyurek and Yavuz [10] in their randomized controlled trial that tried to compare whether differences exist between two viscoelastic foam support surfaces in the development of new PUs found no differences between two different surfaces.

Vanderwee et al. [19] conducted a randomized controlled trial in 447 patients allocated to the intervention group (patient lying on an alternating pressure air mattress) and the control group (patient lying on a viscoelastic foam mattress) in combination with repositioning every four hours. The results indicated that there was no significant difference in the incidence of PUs between the two groups.

The social and economic burden of PU on patients and families as well as on the healthcare system is underlined in many studies conducted in the last decades. This burden is likely to increase and represent reduced quality of life of patients and their families. The development of study protocols along with patient safety data concerning PUs may suggest improvement areas to focus on and extract safe results [20,21].

Some of the limitations of this study were the small studied sample and the number of hospitals participated in the study (only one). Moreover, although patients’ profile assigned to the two groups was comparable, the nursing staff providing the standard preventing care was different between the groups, probably introducing a bias. However, the nursing staff of both ICUs were equally trained and provided the standard PU preventing care following a standard hospital protocol.

## Conclusions

We compared two main mattress types utilized in ICU patients and their effect in preventive and healing process of Pus, and the findings of this study suggest that the Virtuoso mattress is recommended to be used in patients who have no PUs so as to prevent their development, whereas the standard foam mattress should be used in patients who have already developed PUs as it promotes the healing process.

Preventing PUs can be a challenge for healthcare professionals. Preventive strategies such as nursing protocols or guidelines where systematic processes of care have been implemented may reduce overall PU incidence. Prevention of PUs also involves the use of new technology including new supportive surfaces. It is of utmost importance to maintain a culture of PU prevention in a care setting.
